# Accurate deep learning model using semi-supervised learning and Noisy Student for cervical cancer screening in low magnification images

**DOI:** 10.1371/journal.pone.0285996

**Published:** 2023-05-18

**Authors:** Yuki Kurita, Shiori Meguro, Naoko Tsuyama, Isao Kosugi, Yasunori Enomoto, Hideya Kawasaki, Takashi Uemura, Michio Kimura, Toshihide Iwashita

**Affiliations:** 1 Department of Regenerative and Infectious Pathology, Hamamatsu University School of Medicine, Hamamatsu, Shizuoka, Japan; 2 Division of Pathology, Cancer Institute, Japanese Foundation for Cancer Research, Tokyo, Japan; 3 Institute for NanoSuit Research, Preeminent Medical Photonics Education & Research Center, Hamamatsu University School of Medicine, Hamamatsu, Japan; 4 Department of Pathology, JA Shizuoka Kohseiren Enshu Hospital, Hamamatsu, Shizuoka, Japan; 5 Department of Medical Informatics, Hamamatsu University School of Medicine, Hamamatsu, Shizuoka, Japan; Sapienza University of Rome: Universita degli Studi di Roma La Sapienza, ITALY

## Abstract

Deep learning technology has been used in the medical field to produce devices for clinical practice. Deep learning methods in cytology offer the potential to enhance cancer screening while also providing quantitative, objective, and highly reproducible testing. However, constructing high-accuracy deep learning models necessitates a significant amount of manually labeled data, which takes time. To address this issue, we used the Noisy Student Training technique to create a binary classification deep learning model for cervical cytology screening, which reduces the quantity of labeled data necessary. We used 140 whole-slide images from liquid-based cytology specimens, 50 of which were low-grade squamous intraepithelial lesions, 50 were high-grade squamous intraepithelial lesions, and 40 were negative samples. We extracted 56,996 images from the slides and then used them to train and test the model. We trained the EfficientNet using 2,600 manually labeled images to generate additional pseudo labels for the unlabeled data and then self-trained it within a student-teacher framework. Based on the presence or absence of abnormal cells, the created model was used to classify the images as normal or abnormal. The Grad-CAM approach was used to visualize the image components that contributed to the classification. The model achieved an area under the curve of 0.908, accuracy of 0.873, and F1-score of 0.833 with our test data. We also explored the optimal confidence threshold score and optimal augmentation approaches for low-magnification images. Our model efficiently classified normal and abnormal images at low magnification with high reliability, making it a promising screening tool for cervical cytology.

## Introduction

Deep learning (DL) technology has facilitated technological innovations in various fields, including computer vision, natural language processing, and speech analysis. These technologies are also being exploited in the medical field, with devices incorporating DL in endoscopy, radiology, and histopathology being used in actual clinical practice. For example, cytology is a less invasive procedure than histopathology for collecting cells from the lesions of patients directly. Recently, machine learning or DL technologies used for diagnosing smears and liquid-based cytology (LBC) specimens obtained from the cervix have been investigated utilizing digitized glass slide images known as whole-slide images (WSIs) [1–14].

The use of DL technology in cytology has the potential to enable quantitative, objective, and reproducible testing. Training high-accuracy DL models necessitates enough high-quality labeled datasets. However, there are no open-source datasets with sufficient high-quality data in the field of cytology [15]. Therefore, the data for specific purposes must be collected manually, which is time-consuming. In addition, previous studies on cytological evaluations using DL methods were typically meant to classify and detect atypical epithelial cells at a higher magnification, such as 20× or 40× [1–8, 11], resulting in the burden of analyzing as many as 2,000 to 5,000 single cells present in a specimen. A systematic review also revealed that most studies on cytological diagnosis using DL methods had been conducted in experimental settings and have not yet been implemented in clinical practice [15].

Cervical cancer is the fourth most frequent cancer in women worldwide, with an estimated 604,000 new cases and 342,000 deaths in 2020 [16], with low- and middle-income countries accounting for approximately 90% of the cases and deaths [17–20]. For early diagnosis of cervical cancer, its screening using cervical cytology specimens is performed based on the Bethesda System [21]. However, compared to high-income countries, cytology is less extensively employed in low- and middle-income countries due to a lack of healthcare infrastructure and a paucity of cytologists or cytopathologists [18–20, 22, 23].

In the first step of cytology screening, an entire region on a glass slide is observed under low magnification, and the areas where abnormal cells are suspected are observed in detail under higher magnification in the second step. More than 90% of cervical cancer screening specimens are free of atypical cells and are diagnosed as negative for intraepithelial lesion or malignancy (NILM) [24, 25]. In most cases, cytologists or cytopathologists can usually diagnose NILM in the first step. Therefore, establishing a DL model that can evaluate NILM at low magnification, as human diagnosticians usually do, is beneficial for reducing labor and supplementing human resources for screening. However, no studies have been conducted using a DL model for low-magnification observation.

This study aimed to develop a DL model for use at a low magnification that classifies cervical LBC images with less labeled data. We trained a DL model based on a convolutional neural network (CNN) by introducing semi-supervised learning and verified its performance in predicting normal and abnormal images at low magnification. We also evaluated the performance of the model as a screening tool for NILM cases.

## Materials and methods

### Data selection

Cervical specimens were obtained from JA Shizuoka Kohseiren Enshu Hospital patients from October 2020 to October 2021. Only cervical specimens from the patients who did not undergo a hysterectomy or cervical conization were used in the study. Only the first specimen was used for the same patient sampled multiple times during this period. Each specimen was subjected to BD SurePath™ (Becton Dickinson, Inc., Franklin Lakes, NJ, USA) LBC and standard Papanicolaou staining.

Two cytologists (with more than 20 years and 10 years of experience in cytology diagnosis, respectively) and two cytopathologists (each with more than 10 years of experience in cytology diagnosis) diagnosed the LBC specimens. Overall, 140 cases were randomly selected from the above. According to the Bethesda System, 100 of them were diagnosed as intraepithelial lesions: 50 with low-grade squamous intraepithelial lesions (LSIL) and 50 with high-grade squamous intraepithelial lesions (HSIL); the remaining 40 were diagnosed with NILM. The Ethics Review Committees of Hamamatsu University School of Medicine and JA Shizuoka Kohseiren Enshu Hospital approved this study (21–131). We obtained written opt-out consent.

### Data processing and assigning pseudo labels

**Fig 1:**
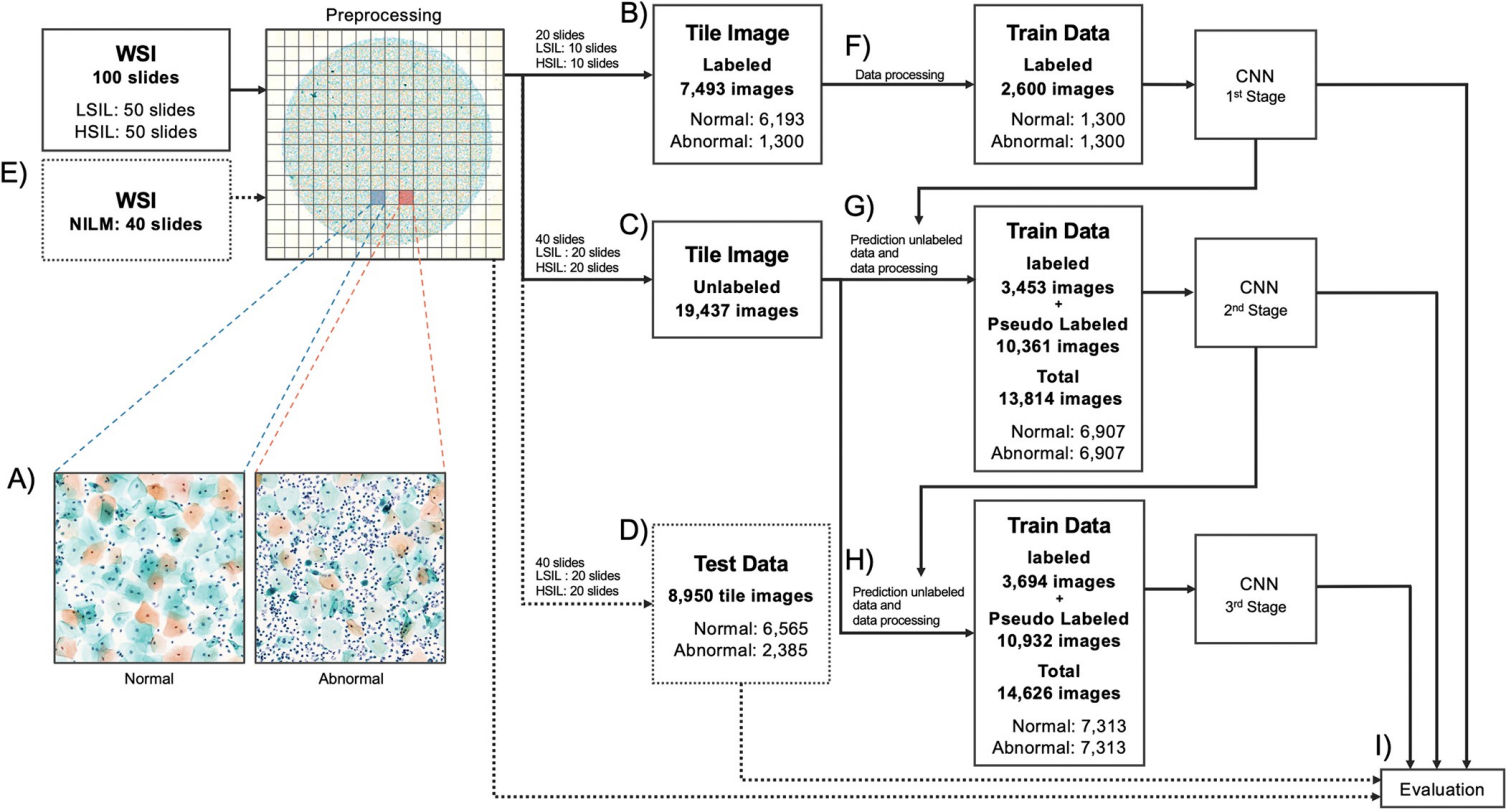
Study method overview. (A) Generate 1024 × 1024-pixel tiled images from WSI. (B) Ten cases each from LSIL and HSIL were randomly selected and manually labeled. (C) Twenty LSIL and HSIL cases were randomly selected, and all tiled images were used as unlabeled data. (D) The tiled images generated from the remaining 20 cases were manually labeled. (E) The test set consisted of all tiled images created from 40 NILM cases. (F) First stage: Tiled images of labeled data labeled normal or abnormal were randomly down-sampled to equalize the number of images and used as training data. (G) Second stage: Using the DL model obtained in the first stage as the teacher model, a pseudo label was assigned to all tiled images in the unlabeled data. The confidence score for the pseudo-label evaluation was calculated and used as the cutoff value. In the second stage, only tiled images with a confidence score of 0.8 or higher were selected. The selected tiled images (pseudo-labeled data) were combined with the labeled data and randomly down-sampled to obtain an equal number of tiled images labeled normal or abnormal. (H) Third stage: Using the DL model obtained in the second stage as the teacher model, pseudo labels were assigned to all tiled images in the unlabeled data. In the third stage, only tiled images with a confidence score of 0.9 or higher were selected. The selected tiled images (pseudo-labeled data) were combined with the labeled data and randomly down-sampled to obtain an equal number of tiled images labeled normal or abnormal. (I) Test data were evaluated at each stage separately, while test cases were only evaluated in the third stage. WSI, whole-slide image; DL, deep learning; LSIL, low-grade squamous intraepithelial lesion; HSIL, high-grade squamous intraepithelial lesion; NILM, negative for intraepithelial lesion or malignancy.

The LBC specimens were scanned at 40× magnification using a whole-slide scanner (NanoZoomer 2.0-HT; Hamamatsu Photonics, Hamamatsu, Japan) and converted into WSIs. The WSIs were divided into small patches of 1,024 × 1,024 pixels (0.92 microns/pixel), called tiled images, equivalent to a 10× objective lens of an optical microscope (Fig 1A). The number of pixels excluding the background of the tiled images was used to determine cell volume per tile, and images with a cell volume of 30% or more were kept for later evaluation.

Fig 1 depicts an overview of the learning pipeline. The CNN model training was divided into three stages. Further, of the 100 WSIs diagnosed as intraepithelial lesions, 10 from LSIL and 10 from HSIL were randomly selected. From these cases, 7,493 tiled images were extracted, and each tile was labeled as normal (when no abnormal cells appeared in the tiled image) or abnormal (when abnormal cells that might be used for cell diagnosis appeared) (Fig 1B). The tiled images labeled as normal were randomly downsampled to equalize the number of the images labeled as abnormal, and 2,600 images were used as training data for training a teacher model in the first stage (Fig 1F). Next, of the remaining 80 WSIs, 20 from LSIL and 20 from HSIL were randomly selected. As an unlabeled dataset, 19,437 tiled images were obtained from these cases (Fig 1C). In the second stage, the teacher model obtained in the first stage was used to assign pseudo labels to the unlabeled data. To select images for pseudo labeling, a confidence score for the prediction of each image was used. Images with a confidence score of 0.8 or higher were selected, and the prediction was applied as a pseudo label on the image. The pseudo-labeled data and 7,493-labeled data were combined; tile images labeled as normal were randomly downsampled to equalize the number of images labeled as abnormal, and 13,814 images were used as training data for training a student model in the second stage (Fig 1G). In the third stage, the model obtained in the second stage was used as a teaching model to assign pseudo labels to the unlabeled images. Images with a confidence score of 0.9 or higher were selected. The same operations as in the second stage were performed.

From the remaining 20 LSIL and 20 HSIL cases, 8,950 tiled images were obtained and were manually labeled as a test set to evaluate the model performance (Fig 1D). From 40 NILM cases, 21,116 tiled images were obtained, and all were labeled as normal (Fig 1E). Ideally, all tiled images obtained from NILM cases should be classified as normal by the model; subsequently, we evaluated the confidence score of each image and abnormal ratio (AR) for each case to assess the model performance as a screening tool for NILM. AR was calculated by dividing the number of images classified as abnormal by the total number of images. A human cytologist manually reviewed images classified as abnormal by the model, and the regions in the image that influenced the prediction were visualized using the Gradient-weighted Class Activation Mapping (Grad-CAM) technique [26].

### CNN training

Noisy Student Training [27] was used as the learning approach in this study. Compared to other semi-supervised learning methods, the Noisy Student method has been widely used for various tasks, including machine-learning competitions. We adopted it because of its ease of implementation. It does not require large amounts of labeled data, and it uses two models: a teacher and a student model. The teacher model is trained on labeled data, following which the model generates pseudo labels for unlabeled data. Then, by combining the labeled and pseudo-labeled data, a student model is trained with noise added to the data. To make the student model equivalent to or better than the teacher model, these training processes are iterated a few times. In this study, two-stage student learning was performed as described above.

EfficientNet [28] was used for a CNN architecture in this study. EfficientNet is a CNN model released in 2019 with a high-performance architecture with fewer parameters than traditional models. The model was pre-trained in ImageNet-1k, which provides eight levels of models (B0−B7) at different scales, and EfficientNet-B3 was used in this experiment.

Table 1 summarizes the number of images and training parameters used to train the model. We did not scale up the model at every stage. For example, we used data balancing without changing the batch size ratio of unlabeled and labeled data because our model was not very large and the dataset was small. Instead of learning from scratch, we used a pre-trained model from ImageNet to make the best use of our relatively restricted computational resources and accelerate the learning process. The dataset was divided using the holdout method and fine-tuning so that no duplicate cases were found in the training and validation data. The training was performed using an RTX A6000 GPU single graphics card (NVIDIA, Santa Clara, CA, USA) with 48 GB memory, with PyTorch serving as the framework.

**Table 1:** Number of images and training parameters used to train the model.

Stage	1^st^	2^nd^	3^rd^
**Training data (%)**	2,013 (77)	11,542 (83)	12,004 (82)
**Validation data (%)**	587 (23)	2,272 (17)	2,622 (18)
**Total**	2,600	13,814	14,626
**Model**	EfficientNet-B3	EfficientNet-B3	EfficientNet-B3
**Pretrained**	ImageNet-1k	ImageNet-1k	ImageNet-1k
**Image size**	300 × 300	300 × 300	300 × 300
**Epoch**	300	250	250
**Batch size**	128	128	128
**Learning rate**	0.001	0.001	0.001
**Criterion**	CrossEntropy	CrossEntropy	CrossEntropy
**Optimizer**	AdamW	AdamW	AdamW
**Weight decay**	0.01	0.01	0.01
**Scheduler**	CosineLR	CosineLR	CosineLR
**Warmup**	−	20 epoch	20 epoch
**Drop Out**	−	0.5	0.5
**Stochastic Depth**	−	0.2	0.2
**Label Smoothing**	0.2	−	−
**Mixup**	−	0.3	0.3
**CutMix**	−	0.3	0.3

### Data augmentation

During training, basic augmentations were performed using the Albumentations library [29] with the following augmentations: VerticalFlip (50%), Rotate (50%), RandomGridShuffle (50%), RandomBrightnessContrast (30%), and RandomGamma (30%). These augmentations were applied to the training data based on established probabilities, and for each epoch, either RandomBrightnessContrast or RandomGamma was applied. In the first stage, only basic augmentation was used to train the teacher model, while in the second and third stages, Mixup [30], CutMix [31], Drop Out [32], and Stochastic Depth [33] were used to train the student model. Augmentation was not applied to the validation data.

### Model evaluation

The area under the curve (AUC), accuracy, and F1 score were calculated at each stage. In addition, the false positive rate (FPR) and false negative rate (FNR) at each stage for the test data were calculated. FPR and FNR were calculated as follows.


$$FPR=\frac{FP}{FP+TN}$$ (1)

$$FNR=\frac{FN}{FN+TP}$$ (2)

FP, TN, FN, and TP stand for false positive, true negative, false negative, and true positive, respectively.

Even images with low prediction probability are classified into one of the two classes in a binary classification task. Therefore, cytologists or cytopathologists should re-confirm images that the DL model classifies with low confidence for screening purposes. We used Youden’s J statistic [34] to determine the confidence score cutoff value. This comprehensive assessment is performed considering sensitivity and specificity, which are important factors in determining diagnostic accuracy. If a calculated confidence score of an image by the model is below the cutoff, the image is classified as abnormal.

Youden’s J statistic (*J*) was calculated as follows:

J=TPR−FPR (3)

$$TPR=\frac{TP}{TP+FN}$$ (4)

TPR represents the actual positive rate.

## Results

### Classification performance

During the training phase, the AUC, accuracy, and F1-score increased with each successive stage, with the highest score in the third stage (AUC: 0.910, accuracy: 0.911, F1-score: 0.910) (Table 2).

**Table 2:** AUC, accuracy, and F1-score results for the validation data.

Stage	1^st^	2^nd^	3^rd^
**AUC**	0.860	0.895	0.910
**Accuracy**	0.862	0.896	0.911
**F1-score**	0.861	0.896	0.910

AUC: area under the curve

**Fig 2:**
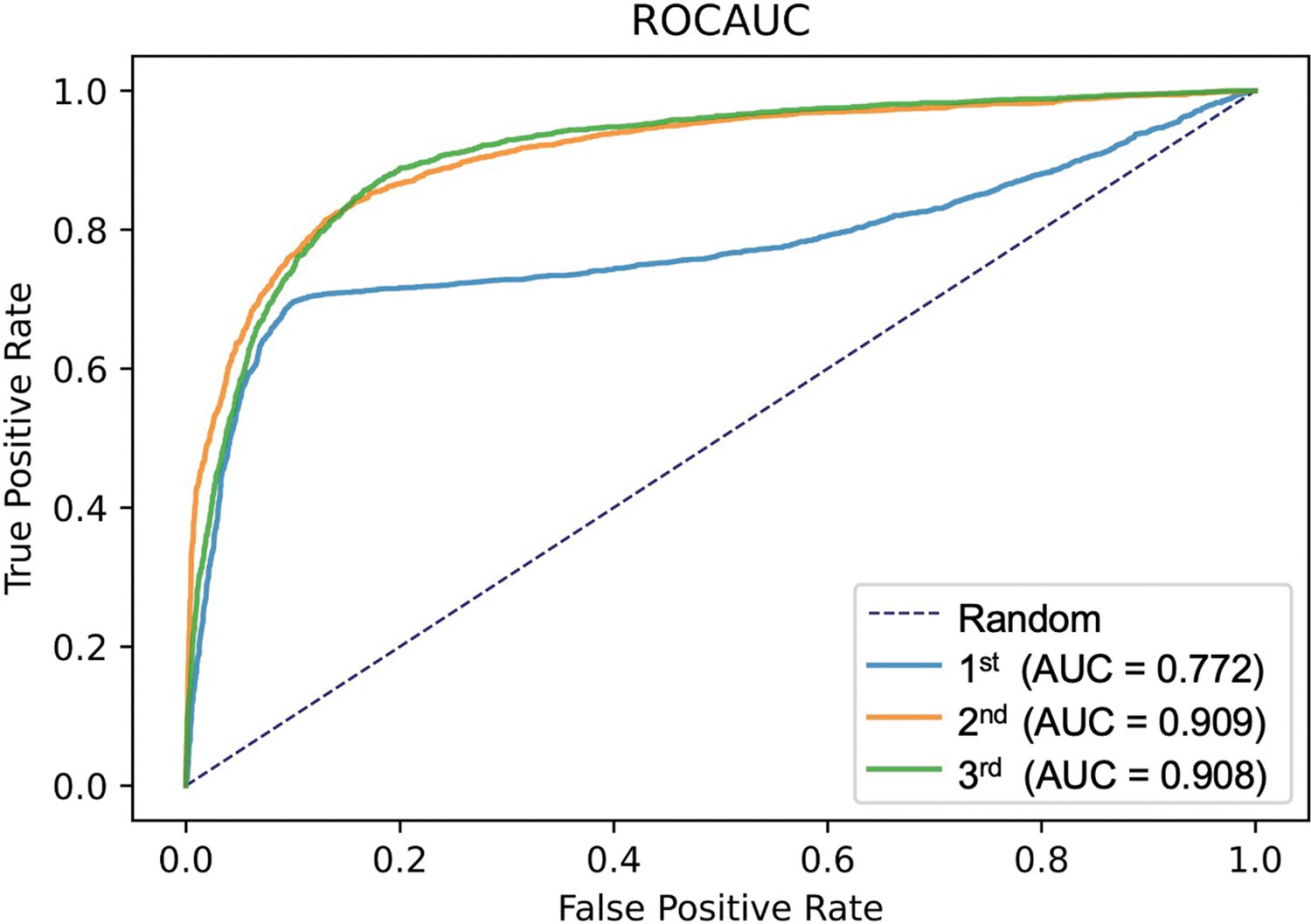
AUC of the ROC curve obtained for the test data. An AUC of 0.909 was achieved in the second stage, and 0.908 was achieved in the third stage. AUC, area under the curve; ROC, receiver-operating characteristic.

For the test data, the receiver operating characteristic (ROC) curve (Fig 2) revealed AUCs of 0.909 and 0.908 for the second and third stages, respectively. The calculated Youden’s J statistic was 0.7, which was used as the confidence score cutoff value. Table 3 shows the confusion matrix. We increased sensitivity while maintaining high specificity and a high F1 score. In addition, cutoff values were adjusted to reduce the FNR, improve sensitivity, and maintain high specificity and a high F1 score (Table 4). This DL model achieved the best performance for the screening process at the third stage.

**Table 3:** Confusion matrix.

Stage		1^st^		2^nd^		3^rd^		3^rd^ (cutoff: 0.7)	
		Predict category		Predict category		Predict category		Predict category	
		**N**	**AN**	**N**	**AN**	**N**	**AN**	**N**	**AN**
**GT**	N	5,880	685	6,156	409	6,082	483	5,913	652
	AN	717	1,668	732	1,653	656	1,729	540	1,845

GT: ground truth, N: normal, AN: abnormal

**Table 4:** Accuracy, FPR, FNR, sensitivity, and specificity results for the test data.

Stage	1^st^	2^nd^	3^rd^	3^rd^ (cutoff: 0.7)
**Accuracy**	0.843	0.873	0.873	0.867
**FPR**	0.104	0.062	0.074	0.099
**FNR**	0.301	0.307	0.275	0.226
**Sensitivity**	0.699	0.693	0.725	0.774
**Specificity**	0.896	0.938	0.926	0.901
**AUC**	0.772	0.909	0.908	–
**F1-score**	0.799	0.829	0.833	0.832

FPR: false positive rate; FNR: false negative rate; AUC: area under the curve

**Fig 3:**
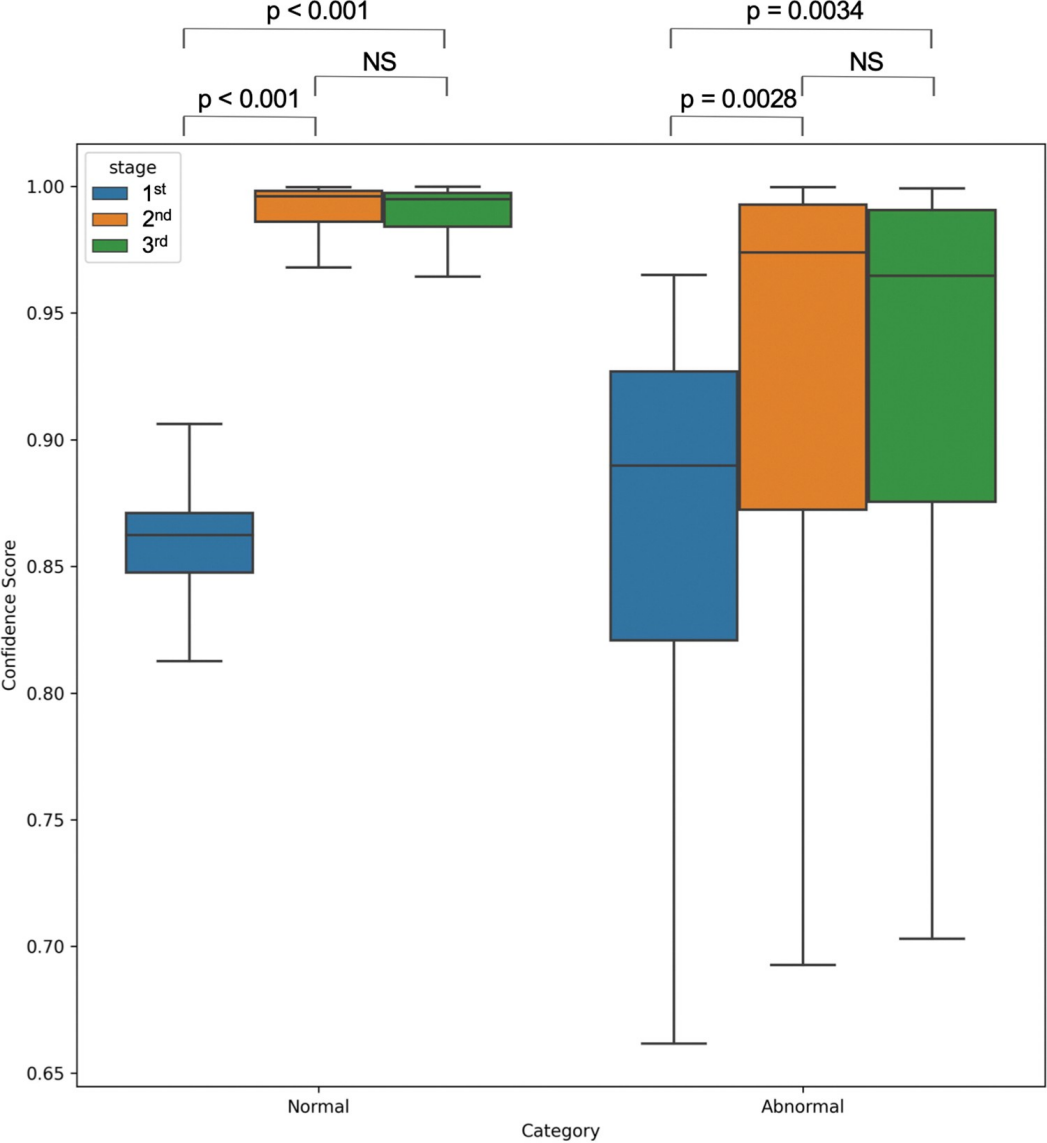
Results of the performance of test data classification. The median confidence score of normal evaluation at each stage was lowest at the first stage (0.862; IQR: 0.848–0.871), varied significantly, and increased markedly at the second stage. There was no difference in standard classification performance between the second (0.996; IQR: 0.986–0.998) and third stages (0.995; IQR: 0.984–0.997). The median confidence score of abnormal evaluation at each stage was lowest at the first stage (0.890; IQR: 0.821–0.927) and increased in the second stage. However, the degree of variation was similar across all stages. The confidence score varied slightly less in the second (0.974; IQR: 0.872–0.991) and third (0.965; IQR: 0.965–0.991) stages. However, there was no difference in the performance for abnormal classification (Fig 2). In the second and third stages, the performance for abnormal classification was slightly lower than that for normal classification. IQR, interquartile range; NS, not significant.

The median confidence score was the lowest at the first stage and increased significantly (normal: p<0.001, abnormal: p = 0.0028) at the second stage for both groups predicted as normal and abnormal (Fig 3). However, no significant difference was observed between the second and third stages. The ranges of confidence score values for the normal group were smaller than those for the abnormal group. The median confidence score for the abnormal classification was slightly lower than that for the normal classification at the second and third stages.

### Evaluation of NILM cases

**Fig 4:**
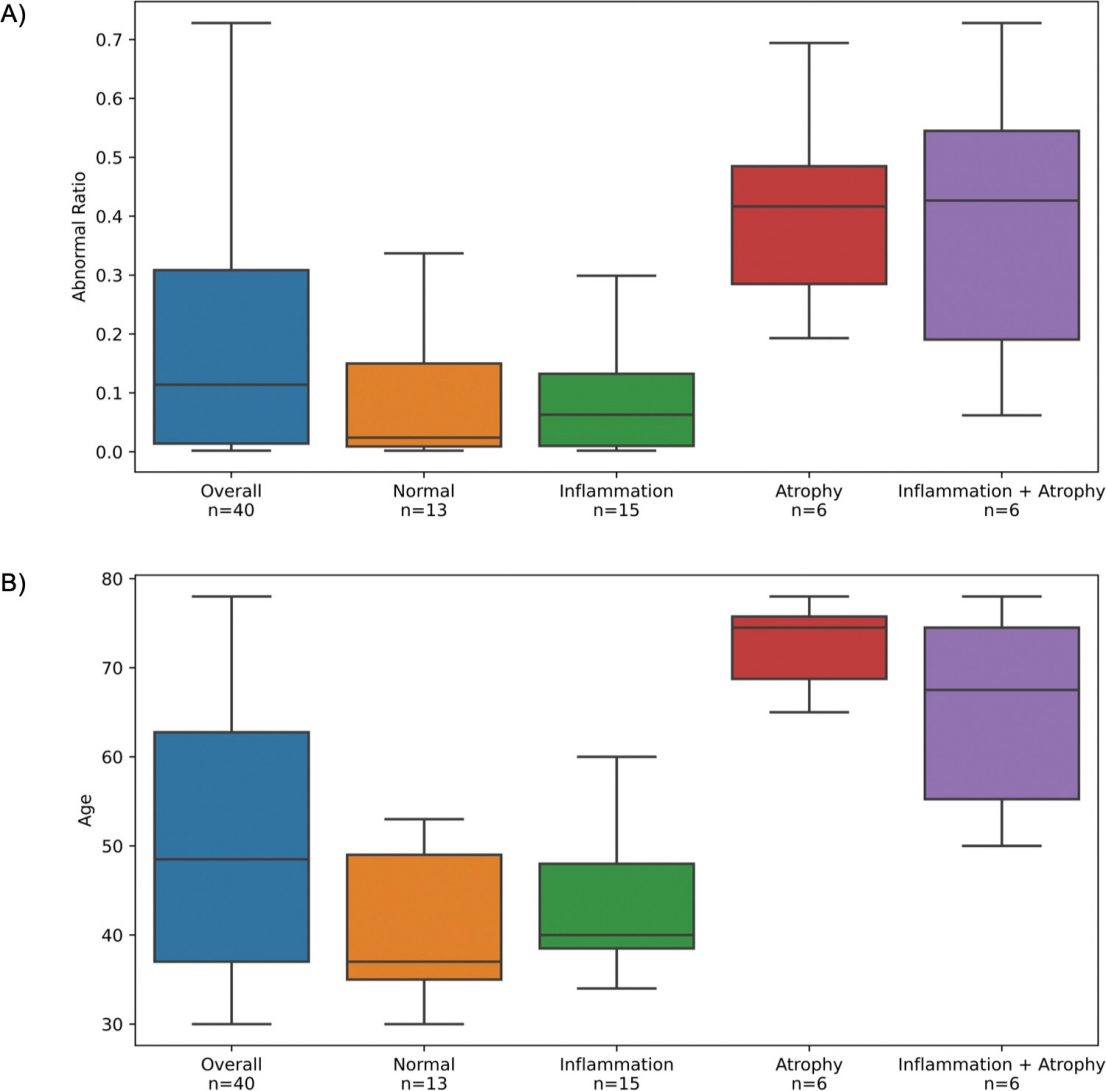
Results of the performance of test case classification. (A) Using the third stage DL model, 40 NILM cases shown in Table 5 were evaluated with a cutoff value of 0.7 for the confidence score. The median AR was 0.114 (IQR; 0.014–0.309). The AR tended to be higher when atrophy-related cellular alterations were present (Fig 5A), with no difference depending on the presence or absence of inflammation. (B) A positive correlation (r = 0.660) between age and AR was observed, and cellular changes associated with atrophy were more common in cases > 50 years. The tiled images contained no abnormal cells that the DL model deemed abnormal. Cellular changes associated with atrophy (Cases 1–7, 9, 12, 15, 21, and 26; Fig 5A), bacterial flora (Cases 14, 22, and 27; Fig 5B), squamous metaplasia (Cases 10 and 17; Fig 5C), endocervical cells (Case 19; Fig 5D), cellular overlap (Cases 8, 11, 13, 14, 16, 18–20, 23–25, and 27; Fig 5E), and cell clusters (Cases 10 and 19; Fig 5F) appeared in the tiled images. DL, deep learning; IQR, interquartile range; AR, abnormal ratio; NILM, negative for intraepithelial lesion or malignancy.

Forty NILM cases were evaluated using the third-stage DL model with a cutoff value of 0.7. Table 5 presents detailed information on the cases and results. The median AR of these cases was 0.114 (IQR: 0.014–0.309) (Fig 4A), and 27 of the 40 cases (67.5%) had an AR of < 0.2. Among 13 cases with AR > 0.2, 7 (53.8%) presented cellular changes associated with atrophy and were over 50 years of age, suggesting that the observed cellular changes were related to aging or postmenopausal changes. Another four cases (30.8) revealed significant cell overlap.

**Table 5:** Detailed information about the test set and evaluation results.

					3^rd^ stage (cutoff: 0.7)		
Case	Age (years)	Inflammation	Atrophy	Total	Normal	Abnormal	Abnormal ratio
**1**	78	+	+	552	150	402	0.728
**2**	74	–	+	507	155	352	0.694
**3**	53	+	+	541	225	316	0.584
**4**	75	–	+	534	273	261	0.489
**5**	76	–	+	548	289	259	0.473
**6**	75	+	+	543	311	232	0.427
**7**	73	+	+	538	309	229	0.426
**8**	37	–	–	536	308	228	0.425
**9**	78	–	+	517	331	186	0.360
**10**	49	–	–	508	337	171	0.337
**11**	49	+	–	546	383	163	0.299
**12**	67	–	+	458	339	119	0.260
**13**	43	+	–	545	435	110	0.202
**14**	30	–	–	545	437	108	0.198
**15**	65	–	+	187	151	36	0.193
**16**	54	+	–	567	464	103	0.182
**17**	78	–	–	427	363	64	0.150
**18**	40	+	–	516	439	77	0.149
**19**	53	–	–	567	492	75	0.132
**20**	34	+	–	619	547	72	0.116
**21**	50	+	+	529	470	59	0.112
**22**	46	–	–	532	492	40	0.075
**23**	40	+	–	546	505	41	0.075
**24**	40	+	–	559	524	35	0.063
**25**	50	+	–	512	480	32	0.063
**26**	62	+	+	438	411	27	0.062
**27**	47	+	–	582	554	28	0.048
**28**	35	–	–	544	531	13	0.024
**29**	40	+	–	538	526	12	0.022
**30**	31	–	–	535	527	8	0.015
**31**	60	+	–	553	547	6	0.011
**32**	36	–	–	558	552	6	0.011
**33**	37	+	–	561	556	5	0.009
**34**	35	–	–	566	561	5	0.009
**35**	37	–	–	549	545	4	0.007
**36**	42	+	–	540	537	3	0.006
**37**	37	+	–	539	537	2	0.004
**38**	53	–	–	528	527	1	0.002
**39**	34	+	–	535	534	1	0.002
**40**	48	–	–	571	570	1	0.002

**Fig 5:**
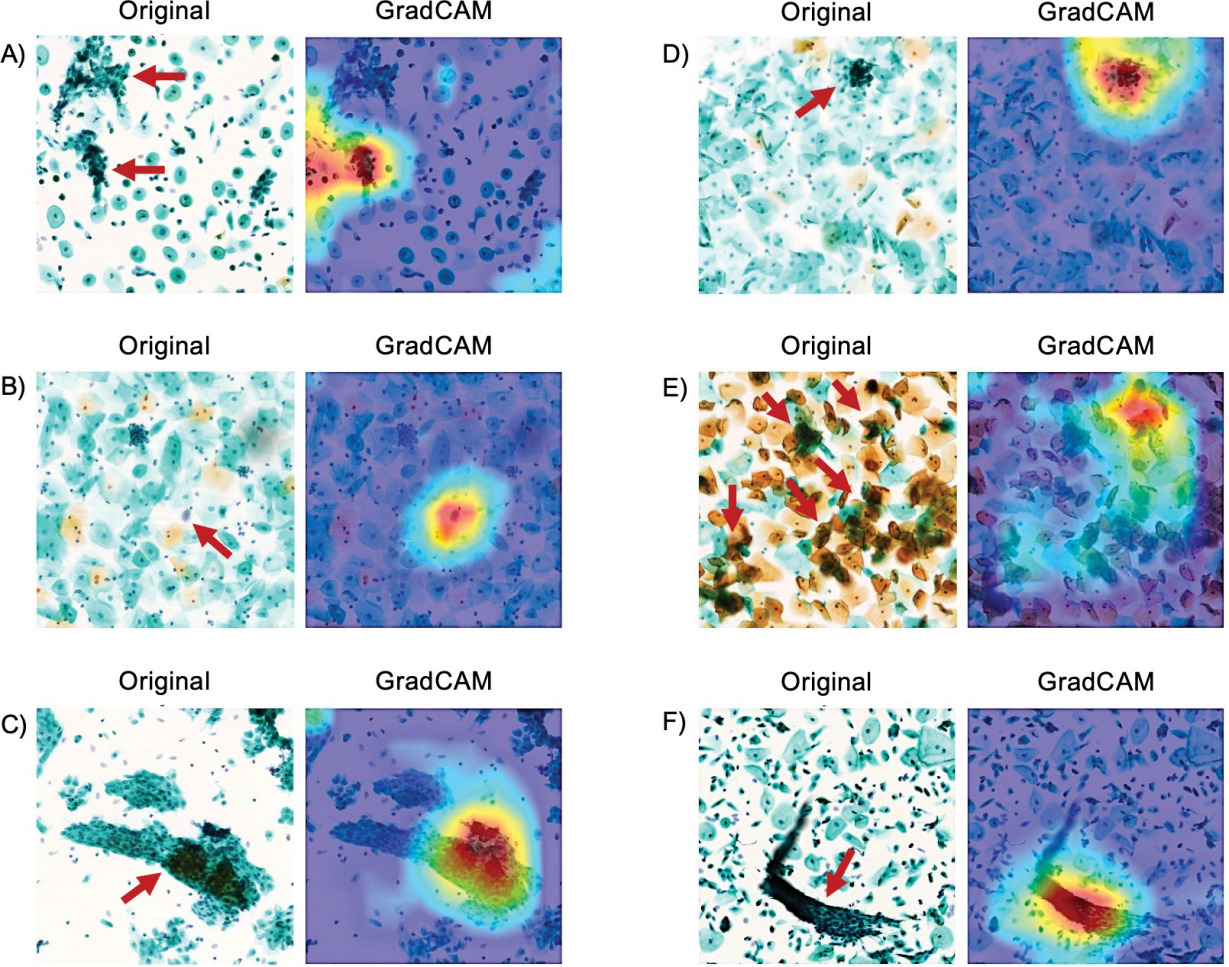
Visualization of images predicted as false positives in the test set and feature maps from Grad-CAM. The DL model focused on these areas when evaluating the image, as highlighted by the areas highlighted in red. Bacterial flora and cell clusters focus on localized areas, while cellular changes with atrophy and cellular overlap focus on a relatively large area. (A) Red arrows indicate cellular changes associated with atrophy (Case 4). (B) Red arrows indicate bacterial flora (Case 22). (C) Red arrows indicate squamous metaplasia (Case 17). (D) Red arrows indicate endocervical cells (Case 19). (E) Red arrows indicate cellular overlap (Case 8). (F) Red arrows indicate cell clusters (Case 10). DL, deep learning.

In the images that the DL model classified as abnormal, cellular changes associated with atrophy (Cases 1–7, 9, 12, 15, 21, and 26; Fig 5A), bacterial flora (Cases 14, 22, and 27; Fig 5B), squamous metaplasia (Cases 10 and 17; Fig 5C), endocervical cells (Case 19, Fig 5D), cellular overlap (Cases 8, 11, 13, 14, 16, 18–20, 23–25, and 27; Fig 5E), and cell clusters (Cases 10 and 19; Fig 5F) were observed. The areas that Grad-CAM heatmaps highlighted in the images confirmed these findings.

## Discussion

Cytologists and cytopathologists can quickly recognize the wide background part and shapes of multiple cells in a cervical cytology smear at low magnification. If no atypical cells are found, the specimen is diagnosed as NILM. In other words, NILM may be diagnosed at low magnification by recognizing the texture of the low-magnification image as a single image and matching it with normal images throughout their careers. Therefore, this study focused on developing a DL model that, at low magnification, identifies images without abnormal cells as being normal, which is the first step in the standard cervical cytology screening process.

**Fig 6:**
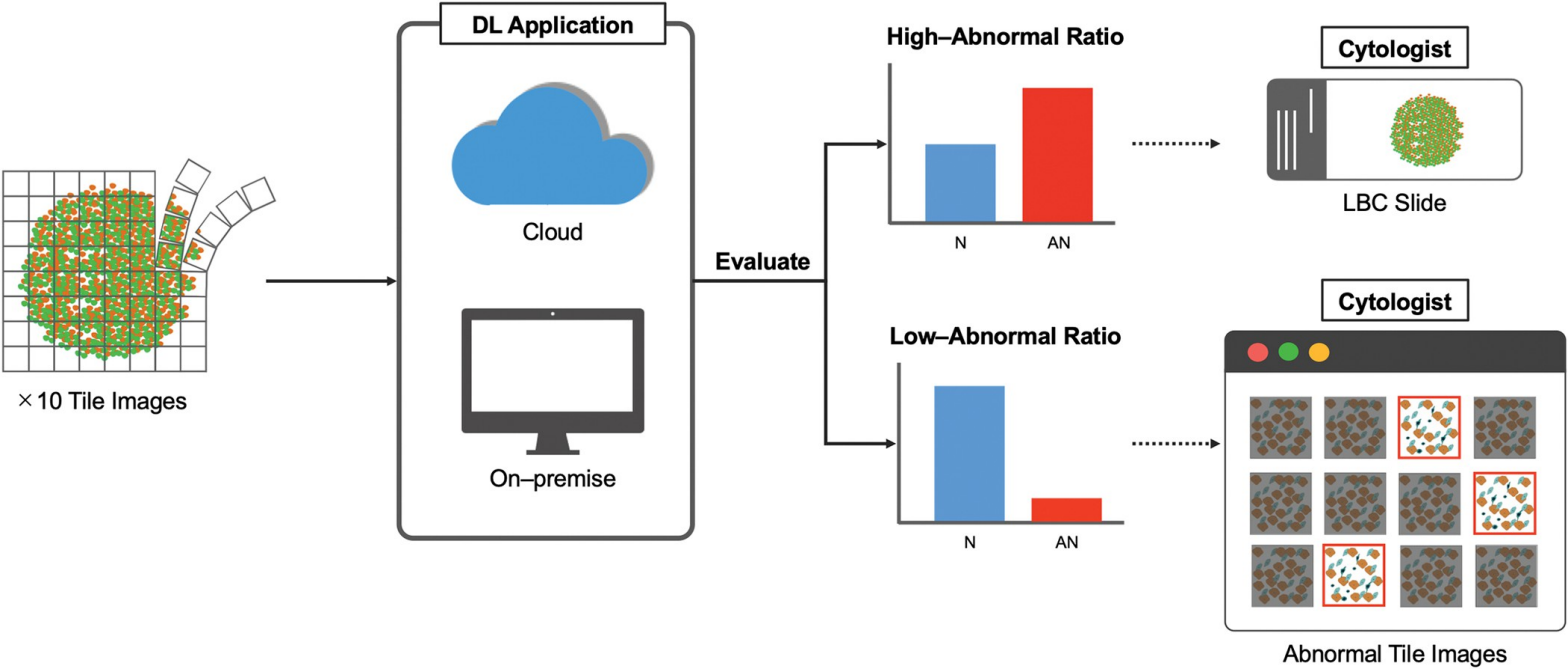
Example of using the developed DL model for screening. The newly designed DL model can be used as a cloud-based web service or on-premises to supplement human resources at a low cost. For example, if the AR in one case is low, the cytologists or cytopathologists check only the tiled images evaluated as abnormal. If the AR is high, the actual specimen is examined under a microscope. AR, abnormal ratio; DL, deep learning.

Previous studies [4, 6–8, 11] focused on diagnosing appeared cells, whereas our DL model aimed at detecting abnormal cells. Noisy Student Training achieved high reliability in classifying cervical cytology images using less labeled data, where only around one-tenth of the total data was used to develop the model. Additionally, because of its high specificity and low AR, our model is suitable as a screening tool for NILM cases. Specimens requiring careful observation include those from older women (>50 years), which tend to present cellular atrophy related to aging or postmenopausal changes, and those showing cell overlapping. The model may regard these changes as abnormal. Further, given the above, a working example can be suggested (Fig 6): when the AR is < 0.2, only the tiled images evaluated as abnormal should be checked by humans, while when the AR is equal to or > 0.2, the physical glass slide should be observed under a microscope. The above operation, which uses the developed DL model, will allow cytologists or cytopathologists to concentrate on cell observation under high magnification and spend more time determining and classifying atypical cells when found.

Cytology is a cost-effective screening method for detecting cervical cancer early. Moreover, even in low- and middle-income countries, the DL model developed in this study may be operated on premises using a compact and inexpensive WSI scanner and a laptop equipped with a GPU. In addition, with the rapid advancement of information and communication technology and the widespread use of mobile devices in low- to middle-income countries, online web applications could be one of the strategies used to engage patients in screening programs [35]. By making the newly developed DL model available as a web application in low- and middle-income countries, it will be possible for cytologists or cytopathologists in these countries to obtain support from their counterparts worldwide. In addition, the use of the application will supplement scarce human resources.

Most previous studies on cervical cytology using DL technology aimed to classify or detect atypical epithelial cells at a single-cell level, where many single cells in the image were classified or detected one by one under high magnification [1–8, 11]. However, depending on the WSI scanner model and the imaging range, a WSI will generate approximately 900 tiled images at 10× and 14,000 at 40×. Further, if a DL model evaluates all the 10× and 40× images, it will take approximately 16 times longer to process images at 40× than at 10×. In other words, developing a DL model that evaluates low-magnification tiled images will significantly reduce WSI processing time.

To introduce DL models into the clinical practice of cytology, the models should be developed to enable image evaluation without difficulty. For example, our dataset contained an average of 4.7 epithelial cells per tiled image at 40× magnification (S1 Fig). Therefore, if each tiled image contains at least one cell, approximately 65,000 (14,000 × 4.7) tiled images (epithelial cells) need to be evaluated. This requires approximately 70 times longer to finish processing a case at 40× magnification than at 10× magnification. This may be one of the reasons why the DL models developed have not been introduced into cytological clinical practice.

New strategies for screening and diagnosing cervical cancer or precancerous lesions have been studied, including the use of artificial intelligence and novel biomarkers. These strategies use various data, such as age, number of sexual partners, age at first sexual intercourse, childbearing history, smoking history, and high-risk HPV genotypes [36, 37]. The model developed in this study exclusively uses cell imaging in cytology. However, there is enormous potential to create a multimodal ensemble model using a large-scale model, including the model we developed and other essential data besides images, for various purposes, including predicting the occurrence and recurrence risk, in addition to cervical cancer diagnosis. Furthermore, the development of multimodal models using diverse data has great potential for various applications, such as difficult treatment decision-making, determining follow-up frequency, and making decisions about the use of low-invasive surgery, which requires a wide range of operations [38–42]. However, the datasets now available are limited, and the approaches are diverse. Further, given disparities in accuracy due to racial or cultural diversities, it may be necessary to construct a large-scale global dataset. Artificial intelligence has also been developed for rapid WSI diagnosis; however, validation is limited, and more testing is necessary using benchmark datasets with large computing resources, datasets, and algorithm development methodologies.

Adding RandomGridShuffle to the primary augmentation contributed to improved performance (S2 Fig). In general, an augmentation that swaps patch images, such as RandomGridShuffle, is rarely used because it significantly changes the structure of the image and is thus used in a minimal range of applications [43, 44]. To our knowledge, this is the first experiment in the cytology field to use RandomGridShuffle, and its application to low-magnification cytology images was successful. We used the RandAugment automated data augmentation method [45], which is a robust data augmentation method that applies rotations and transforms to image data while searching for suitable parameters for augmentation. However, it did not improve the performance of our model. RandAugment searches for augmentations that process existing data while preserving the meaning through geometric and color space manipulations. Nonetheless, it excludes augmentations that change the structure of the image. Therefore, because the texture of each image appears similar, low-magnification tiled images may create over-fitting. Therefore, we used RandomGridShuffle, an augmentation that generates artificial data from known data by dividing the image into *n* × *n* patch images and randomly replacing them. Further, while a larger *n* result leads to more information loss and lower accuracy [46], it was possible to maintain local texture as long as *n* was not too large (S2 Fig). We assumed that RandomGridShuffle brought diversity to low-magnification tiled images in our data and suppressed over-fitting.

LBC can generate uniformly distributed cells on slides and reduce cellular artifacts, and it is challenging to reduce false positives, which can occur in normal images due to overlapping cells. The use of Z-Stack [47] and generative adversarial networks (GAN) [48, 49] have also been investigated as techniques to minimize the effects of cellular overlap in cytology images. However, Z-Stack has the technical problem of long scan times during WSI creation and substantial WSI data volume [50], and GAN requires a large amount of data, making it an arduous task. Given the difficulty of the analysis technique, improving LBC specimen preparation techniques and minimizing cellular overlap are required.

## Conclusions

In this study, we used semi-supervised learning to develop a DL model for screening cervical cytology specimens. By integrating Noisy Student Training, which reduces the amount of labeled data needed for training, we were able to achieve an AUC of 0.910 for the test data. Furthermore, we found the optimal threshold for confidence score and the optimal augmentation for low-magnification tiled images. The DL model we have developed is expected to be utilized in screening work for cervical cytology, as it can be used to evaluate normality and abnormality in low-magnification tiled images accurately.

## Supporting information

S1 Fig Number of epithelial cells contained per 40× tiled image. The 40× tiled image contains an average of 4.7 epithelial cells per image.
(TIF)

S2 Fig Change in the AUC score depends on the presence or absence of RandomGridShuffle, RandAugment, and patch size. The AUC was lower when RandomGridShuffle was not applied or when RandAugment was applied, and changing patch size caused changes in scores. The highest AUC was obtained with a patch size of 4 × 4. RandomGridShuffle was set to be applied to training data with a probability of 50%. AUC, area under the curve.
(TIF)
