# Supplementary figures and images for: Accurate deep learning model using semi-supervised learning and Noisy Student for cervical cancer screening in low magnification images
Source: PLoS One. 2023 May 18;18(5):e0285996. doi: 10.1371/journal.pone.0285996 (PMC10194854; doi:10.1371/journal.pone.0285996)

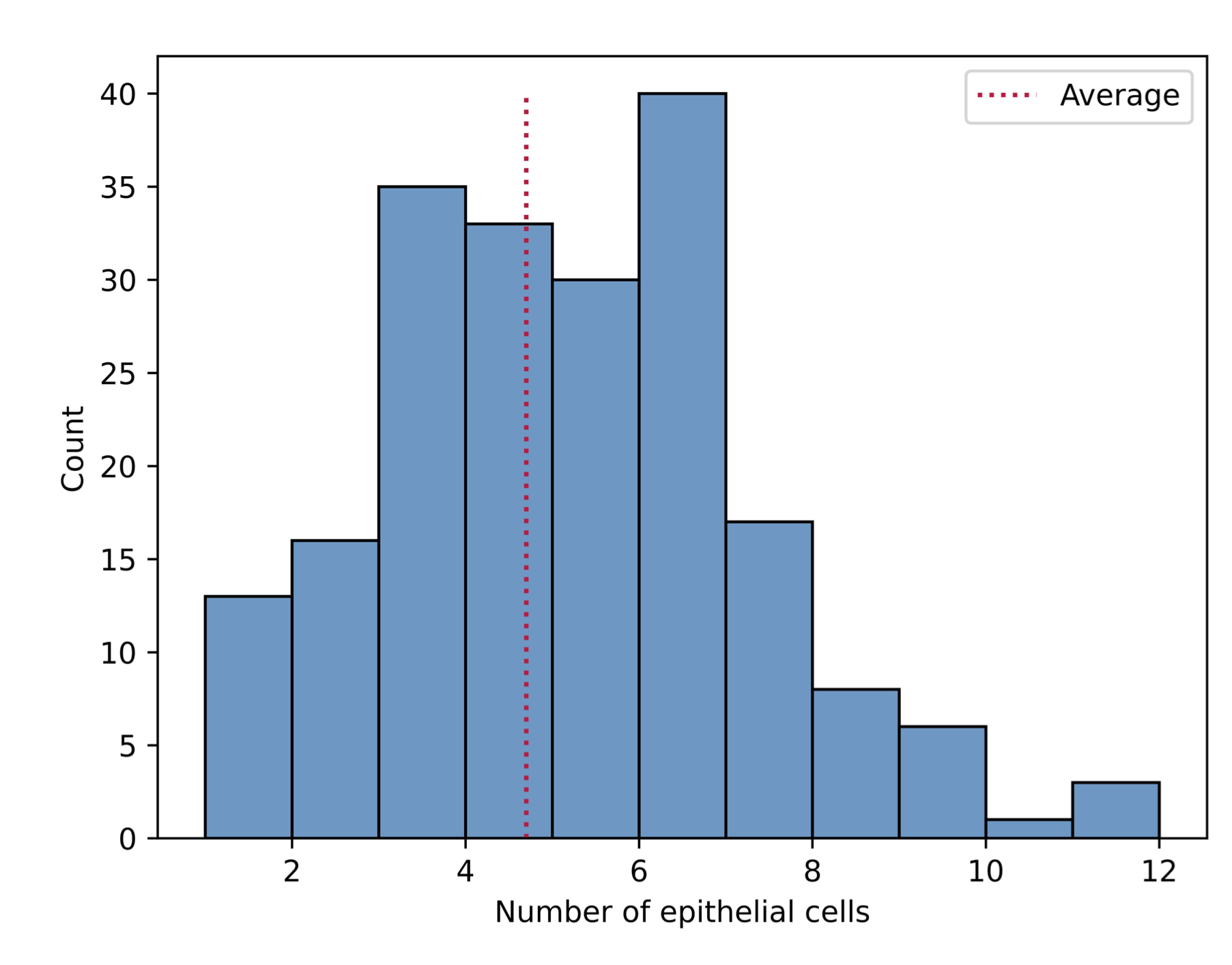

Supplement: S1 Fig — The 40× tiled image contains an average of 4.7 epithelial cells per image. (TIF) [file pone.0285996.s001.tif]

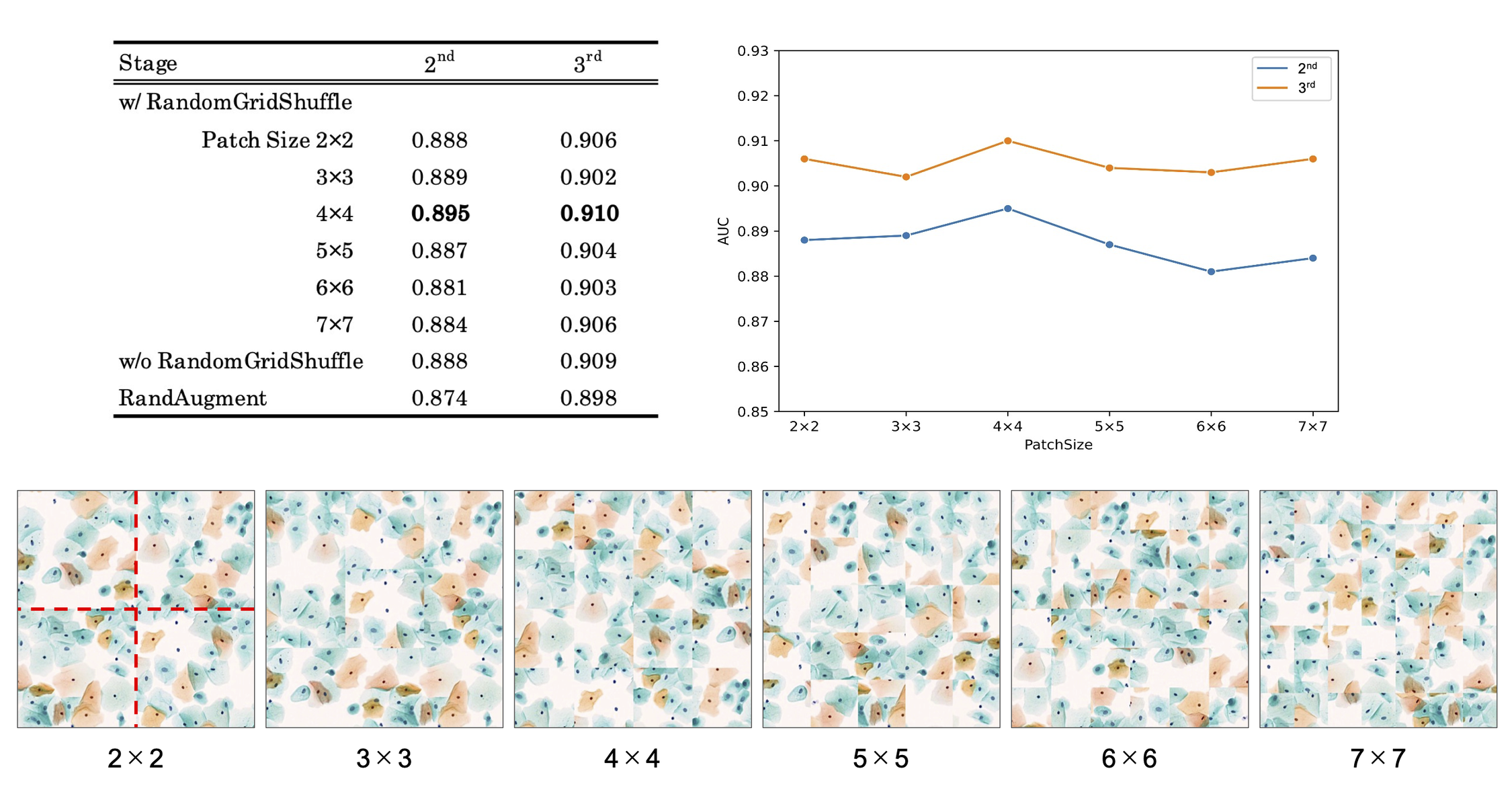

Supplement: S2 Fig — The AUC was lower when RandomGridShuffle was not applied or when RandAugment was applied, and changing patch size caused changes in scores. The highest AUC was obtained with a patch size of 4 × 4. RandomGridShuffle was set to be applied to training data with a probability of 50%. AUC, area under the curve. (TIF) [file pone.0285996.s002.tif]
